# Meta-analysis of the efficacy of rituximab in the management of cryoglobulinemic vasculitis

**DOI:** 10.3389/fmed.2025.1591366

**Published:** 2025-08-29

**Authors:** Ling Zhou, Jianxia Dong, Zehui Deng, Shaojuan Wang, Mengjie Fu, Jingjing Peng, Jian Zhang

**Affiliations:** Department of Pharmacy, West China Hospital, Sichuan University, Chengdu, China

**Keywords:** rituximab, cryoglobulinemic vasculitis, vasculitis meta-analysis, clinical efficacy, anti-CD20

## Abstract

**Introduction:**

A primary goal of this study was to systematically assess the efficacy of rituximab (RTX) in treating cryoglobulinemic vasculitis (CV).

**Methods:**

A prospectively registered meta-analysis was conducted to examine eligible randomized controlled trials (RCTs) or cohort studies through searches across PubMed, Embase, Cochrane Library, and Web of Science, with a search period up to February 12, 2025. Data analysis was conducted utilizing STATA 16.0.

**Results:**

Incorporating data from 12 studies involving 287 patients, CV patients who received RTX therapy demonstrated notable complete clinical response outcomes (Rate = 0.67, 95% confidence interval (95%CI): 0.61, 0.73) and a good clinical response rate. In addition, patients showed significant relief in symptoms such as skin purpura and skin ulcer (Rate = 0.92, 95%CI: 0.86,0.98). The meta-analysis findings indicated a notable enhancement in serum C4 levels in CV patients following treatment (mean difference (MD) = 0.06, 95%CI: 0.04, 0.07), both at 6-month (MD = 0.07, 95%CI: 0.05, 0.09) and 12-month (MD = 0.07, 95%CI: 0.03, 0.11) follow-ups. These findings suggest a gradual improvement in the underlying condition. The levels of IgM were significantly reduced following treatment (MD = −0.48, 95%CI: −0.65, −0.31), both at 6-month (MD = −1.05, 95%CI: −1.57, −0.52) and 12-month (MD = −0.59, 95%CI:−0.80, −0.38) follow-ups. The levels of cryoglobulin were also decreased following treatment (MD = −0.53, 95%CI: −0.80, −0.26), both at 6-month (MD = −0.67, 95%CI: −0.99, −0.35) and 12-month (MD = −0.67, 95%CI: −1.15, −0.19) follow-ups. Similarly, rheumatoid factor (RF) levels significantly decreased after treatment (MD = −318.20,95%CI:−364.66,−271.73) and remained low at the 6-month follow-up (MD = −287.78, 95%CI:−511.58,−63.97).

**Discussion:**

The meta-analysis supports the favorable clinical efficacy of rituximab in the management of CV patients. However, further validation through additional high-quality RCTs is warranted to solidify its effectiveness.

**Systematic review registration:**

https://www.crd.york.ac.uk/PROSPERO/view/CRD42024565790.

## Introduction

Cryoglobulinemic vasculitis (CV) is a systemic condition characterized by inflammation of small blood vessels, driven by the clonal expansion of B-cell clones that produce pathogenic immune complexes known as cryoglobulins. Frequently, it arises as a secondary complication of hepatitis C virus (HCV) infection, autoimmune disorders, and hematological malignancies (1). Cryoglobulinemia is primarily classified into type I and type II/III. Type II and III cryoglobulinemia are typically categorized as mixed cryoglobulinemia (MC), frequently linked to chronic viral infections (HCV, HBV and others) or autoimmune diseases. Furthermore, they are also associated with lymphoproliferative diseases or they are defined as essential, as no underlying cause is found EMC (essential mixed cryoglobulinemia, EMC) (2). Studies have estimated that the incidence of type I cryoglobulinemia in patients with clinical manifestations is about 1/100000, and it is more common in individuals aged 45–65 (3). Among patients with HCV-related mixed cryoglobulinemia, 20–30% present with significant organ damage, and 2–5% develop rapidly progressive and life-threatening vasculitis (4). The recurrence rate remains relatively high. Results indicated that 14 out of 36 patients (39%) experienced a relapse of CV within days to 19 months (with an average of 6.7 months) after their last rituximab infusion. Among these, 13 patients were infected with HCV, and 1 patient was not HCV-infected (5). The pathogenesis of CV is currently not clear. Due to the varied clinical manifestations, high recurrence rates, and therapeutic resistance, the management of CV remains a significant challenge.

At present, traditional treatment methods for CV mainly include glucocorticoids, antiviral therapy, immunosuppressants, and plasma exchange (6). However, these methods frequently prove inadequate for clinical use due to their suboptimal efficacy, considerable adverse effects, or requirement for frequent administration. Rituximab (RTX) is a genetically engineered chimeric anti-CD20 monoclonal antibody and has high efficacy in clearing B cells *in vivo* (7). Multiple clinical studies have shown that (8–12) RTX has significant efficacy in treating CV, with a high clinical remission rate. Furthermore, RTX also demonstrates significant clinical value as a first-line treatment for HCV-associated CV with severe organ involvement, such as nephropathy and peripheral neuropathy (13). The Kidney Disease: Improving Global Outcomes (KDIGO) guidelines recommend RTX in combination with antiviral therapy for HCV-associated cryoglobulinemic glomerulonephritis (14). RTX is also used to manage symptoms related to cryoglobulinemia, such as progressive nephropathy (15). Related research has further validated that RTX treatment is contributes to the recovery of renal function (16).

Existing studies have demonstrated the efficacy of RTX in the treatment of CV. However, due to the lack of comprehensive analysis among these studies, there is no consensus. Consequently, a meta-analysis is required to rigorously assess the real-world performance of RTX in CV. This analysis is intended to strengthen the evidence base for the clinical use of RTX in managing CV.

## Methods

The meta-analysis adhered to the guidelines outlined in the Preferred Reporting Items for Systematic Reviews and Meta-Analyses (PRISMA) (17). The research protocol was recorded in the International Prospective Register of Systematic Reviews (PROSPERO, CRD42024565790).

### Search strategy

A search of the English literature within each database (PubMed, Embase, Cochrane Library, and Web of Science) was conducted from its inception until February 12, 2025. During this process, a combination of subject headings and textwords was applied. The medical subject headings included: Rituximab, Vasculitis, Cryoglobulinemic, Cryoglobulinemia. The detailed search procedure is outlined in Supplementary Tables S1–S4.

### Inclusion and exclusion criteria

To draw reliable conclusions regarding the effectiveness of RTX in managing CV, high-quality research literature meeting the inclusion and exclusion criteria was systematically sought. The inclusion criteria adhered to the Population, Intervention, Comparison, Outcomes and Study (PICOS) guidelines (18). Studies that met the following criteria were incorporated into this meta-analysis:

#### Participant

Individuals diagnosed with CV.

#### Intervention

Patients treated with RTX.

#### Outcome

Rate of complete clinical response and alleviation of symptoms (skin purpura and skin ulcers) post-treatment; changes in laboratory markers [IgM, C4, cryoglobulin, and rheumatoid factor (RF)] from baseline to 6 months and 12 months post-treatment, and during follow-up.

### Study type

RCTs or cohort studies.

Exclusion criteria included: (1) Studies lacking available outcome measures; (2) Animal or cell trials, case studies, scientific experimental plans, comments, letters, editorials, and conference abstracts; (3) Studies without full-text access.

### Data extraction

Retrieved articles were imported into EndNote20. Two researchers (Zhou and Deng) independently screened titles and abstracts against the inclusion and exclusion criteria. A subsequent full-text review was then conducted for further assessment. Inconsistencies were addressed through deliberation or seeking advice from a third researcher (Wang). Utilizing Excel 2016, the two researchers autonomously gathered data from the selected studies, including information on the primary author, publication year, region, experimental design, diagnostic criteria, patient type, number of cases, gender, age, and outcome measures.

### Quality assessment

Due to the inclusion of both RCTs and cohort studies, the Cochrane Risk of Bias Tool (RoB2.0) (19) was employed to assess the risk of bias across six domains: bias stemming from the randomization process, deviations from intended interventions, missing outcome data, outcome measurement accuracy, selective reporting, and other potential sources of bias. The risks were categorized into three groups: “low risk,” “high risk,” or “some concerns” across the aforementioned six domains. The results of the assessments are presented in risk of bias graphs. The Newcastle–Ottawa Scale (NOS) was utilized to evaluate the quality of the included cohort and case–control studies, assessing both study quality and the risk of bias (20). The assessment encompassed the following items: cohort selection (including representativeness of the exposed cohort, selection of the non-exposed cohort, confirmation of exposure, and exclusion of subjects with the disease of interest at study onset), comparability, and outcome indicators (including method of outcome ascertainment, follow-up period, and integrity of follow-up). The quality of the included studies was assessed using the NOS star rating system, with a maximum score of 9 stars. Studies with a score of 6 or more were deemed to be of moderate quality. Only studies scoring above 6 will be included in the meta-analysis. The evaluation results will be presented utilizing the NOS scoring system. The quality of each article was assessed independently by two investigators. Inconsistencies were addressed through deliberation or seeking advice from a third researcher.

### Statistical analysis

Data were analyzed using STATA 16.0. Continuous data were evaluated by computing the standardized mean difference (SMD). Odds ratios (ORs) were adopted to analyze the dichotomous data, and the overall results were represented using 95% confidence intervals (95%CIs). Study heterogeneity was evaluated utilizing Cochran’s Q test and I^2^ statistic. Given the substantial heterogeneity indicated by an *I*^2^ exceeding 50%, a random-effects model was employed in sensitivity analysis to explore potential sources of heterogeneity. When *I*^2^ was less than 50%, a fixed effects model was utilized to assess the effect size (21). A series of meta-analyses were performed on eligible literature to assess the effectiveness of RTX in managing CV (alleviation of clinical symptoms and improvement of laboratory indicators). Publication bias was evaluated utilizing funnel plots and Egger’s test. A two-tailed *p*-value < 0.10 was deemed statistically significant when assessing heterogeneity. All statistical differences with *p* < 0.05 were considered notable.

## Results

### Article retrieval and screening

**Figure 1:**
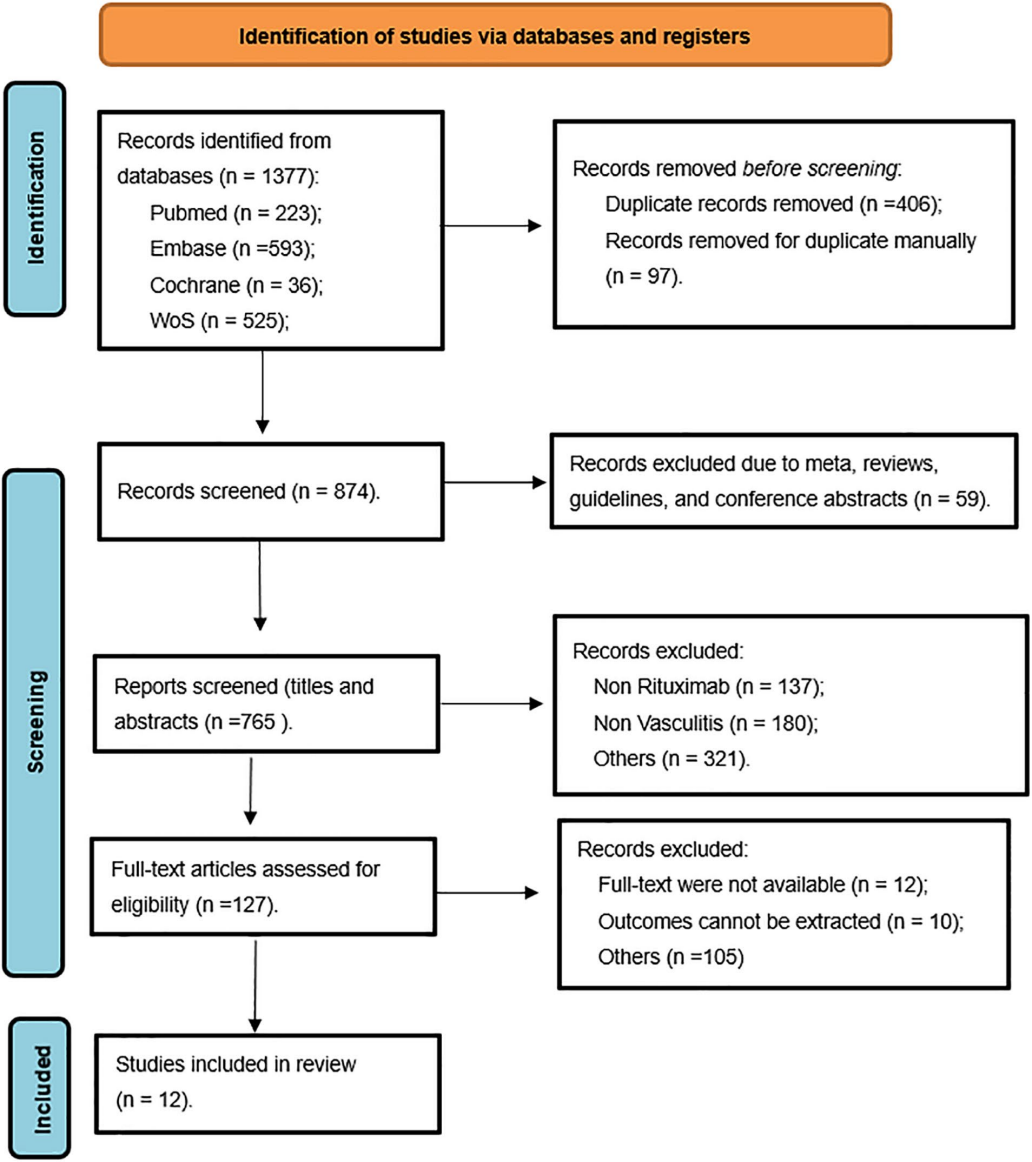
Flow diagram of the study identification and selection procedure.

A search process is visually depicted in Figure 1. Of the initial 1,377 articles retrieved, 503 duplicates were removed. Subsequently, 109 records, including conference abstracts, clinical trial registration records, meta-analyses, and reviews, were excluded. After preliminary reviewing of the titles and abstracts, 638 articles were removed. Based on a full-text review and strict application of the inclusion and exclusion criteria, 12 articles were selected for the final analysis (8, 10, 11, 22–30).

### Baseline characteristics of included studies

The 12 studies, conducted in France (*n* = 11) and the USA (*n* = 1), included a total of 287 patients. The study design comprised 7 cohort studies (11, 22, 24–26, 29, 30) and 5 RCTs (8, 10, 23, 27, 28). Regarding diagnostic criteria, two studies included patients who had not received antiviral drugs, interferon, or immunosuppressants (8, 24). One study included patients who were either non-responsive to or intolerant of interferon-alpha and ribavirin (27). The remaining studies did not specify diagnostic criteria that limited prior patient therapy. The patient populations primarily consisted of CV patients with HCV infection. Among these studies, one recruited CV patients without HCV infection (30). Another study did not specify the MC type (25), while two studies recruited type II MC patients (23, 29). A combination of RTX and other drugs is frequently applied in RCTs (Pegylated interferon *α*/ribavirin) (8, 10, 27, 28). However, these studies frequently lack a blank control for RTX. Only one study included a blank control for RTX (23). Therefore only data from groups that received RTX were analyzed in the RCTs. RTX treatment efficacy was assessed at varying time points across studies: 1 month (*n* = 3) (25, 27, 29), 2 months (*n* = 1) (23), and 3 months (*n* = 4) (10, 11, 26, 28). Table 1 displays the fundamental characteristics of the articles enrolled in the analysis.

**Table 1:** Baseline characteristics of enrolled articles.

Author, Year	Type of study	Country	Diagnostic criteria	Patient type	Number of included cases (RTX)	Sex (M/F)	Age (years)	Outcome indicators
Basile et al. (2021) (22)	Cohort study	France	NA	HCV or HBV associated MC vasculitis	34	8/26	35–82	Complete clinical responder
Dammacco et al. (2010) (8)	RCT	France	(1) Detection of serum cryoglobulins; (2) positivity for anti-HCV antibodies and polymerase chain reaction (PCR)–based assay to detect HCV RNA in serum; (3) liver biopsy showing chronic hepatitis, performed within 3 months from enrollment; (4) negativity for hepatitis B surface antigen and human immunodeficiency virus; (5) no previous administration of IFNs or immunosuppressive drugs.	HCV-related MC	22	7/15	51–68	IgM, skin: purpura, ulcers, C4, RF, probability of complete response
Vita et al. (23)	RCT	France	(1) Had serum cryoglobulins at study entry; (2) the study patients had manifestations of severe active CV; (3) in patients with HCV related CV, study inclusion implied that therapy with antiviral agents with interferon plus ribavirin had failed, had been poorly tolerated, or was considered to be contraindicated; (4) negative for antibodies against human immunodeficiency virus and hepatitis B virus	Patients with CV who had type II cryoglobulins	28	4/24	62.85 ± 11.36	Skin ulcers, RF, C4
Quartuccio et al. (2015) (24)	Cohort study	France	(1) HCV-positive; (2) untreated with antiviral drugs during the published 24-month study	HCV-related CV	30	6/24	37–78	Cryoglobulins, skin ulcers
Roccatello et al. (2016) (25)	Cohort study	France	NA	MC	31	13/18	36–80	IgM, RF, C4, skin ulcers, complete clinical responder
Saadoun et al. (2008) (10)	Cohort study	France	(1) Cryoglobulin > 0.05 g/L on at least two occasions, which was associated with purpura, and arthralgia with renal and/or neurological involvement; (2) positive for HCV RNA.	HCV-related CV	16	NA	NA	Cryoglobulin, C4, IgM, RF, skin, complete response
Saadoun et al. (2010) (11)	Cohort study	France	(1) Cryoglobulin > 0.05 g/l on at least two occasions, which was associated with purpura, and arthralgia with renal and/or neurological involvement; (2) Positive for HCV RNA.	HCV-related CV	38	12/26	58 ± 11.8	Complete clinical responder, cryoglobulin, C4, skin
Saadoun et al. (2008) (10)	RCT	France	(1) Cryoglobulin > 0.05 g/l on at least two occasions; (2) positive for HCV RNA.	HCV-related CV	21	NA	29–77	Cryoglobulin, complete clinical responder
Sneller et al. (2012) (27)	RCT	U. S. A	(1) The presence of active manifestations of HCV-associated cryoglobulinemic vasculitis, as evidenced by one or more of the following: cutaneous vasculitis, peripheral neuropathy, or glomerulonephritis; (2) treatment with interferon-a and ribavirin failed to induce a response or who could not tolerate this therapy	HCV-related CV	12	NA	NA	Complete clinical responder, cryoglobulin
Terrier et al. (2009) (28)	RCT	France	(1) Cryoglobulin > 0.05 g/l on at least two occasions; (2) positive for HCV RNA.	HCV-related CV	12	8/4	44–77	Cryoglobulin, C4, RF, IgM, complete clinical responder, skin
Zaja et al. (2003) (29)	Cohort study	France	NA	Type II MC	15	4/11	NA	IgM, RF, cryoglobulin, C4, skin, complete clinical responder
Terrier et al. (2012) (30)	Cohort study	France	(1) Type II or type III MC after detection and immunochemical typing; (2) systemic vasculitis; (3) diagnosis of CryoVas between January 1995 and July 2010. (4) The exclusion criteria were the presence of anti-HCV and anti-HIV Abs and hepatitis B surface Ag	CV for non-HCV infection	28	NA	NA	Complete clinical responder

### Methodological quality assessment of articles enrolled

**Figure 2:**
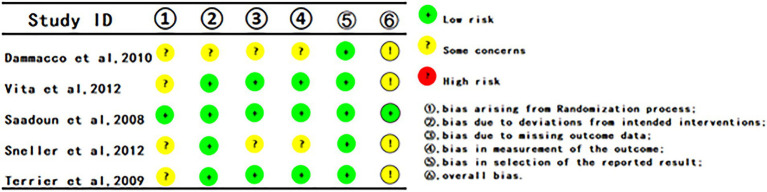
Risk of bias assessment.

Figure 2 illustrates the risk of bias assessment results for the five included studies. Regarding bias related to randomization, four articles (8, 23, 27, 28) were classified as having some concerns due to the absence of concealment assessment, while the remaining one was classified as having a low risk of bias. All articles related to deviations from intended interventions, missing outcome data, and outcome measurement were categorized as having a low risk of bias. All articles that showed no evidence of selective reporting were deemed to be at low risk. No other sources of bias were identified in RCTs, all of which were deemed to be at low risk. Overall, the included RCTs exhibited a low risk of bias.

The seven included cohort studies underwent a quality assessment encompassing three key domains: selection of the cohort, comparability, and outcome measurement. All scores exceeded 6 points, suggesting relatively high quality among the included cohort studies. The assessment outcomes are detailed in Table 2.

**Table 2:** Methodological quality of prospective case–control research based on the NOS.

Included studies	Study population selection	Comparability	Exposure or results	Levels
Basile et al. (2021) (22)	☆☆	☆☆	☆☆☆	7☆
Quartuccio et al. (2015) (24)	☆☆	☆	☆☆☆	6☆
Roccatello et al. (2016) (25)	☆☆☆☆	☆☆	☆☆☆	9☆
Saadoun et al. (2008) (10)	☆☆☆	☆☆	☆☆☆	8☆
Saadoun et al. (2010) (11)	☆☆	☆☆	☆☆☆	7☆
Zaja et al. (2003) (29)	☆☆☆	☆☆	☆☆☆	8☆
Terrier et al. (2012) (30)	☆☆☆	☆☆	☆☆	7☆

Stars are used to denote scores. ☆corresponds to one point, ☆☆correspond to two points, ☆☆☆correspond to three points, ☆☆☆☆correspond to four points.

## Results of meta-analysis

### Primary outcome: clinical efficiency

#### Complete clinical response and alleviation rate of symptoms such as skin purpura and skin ulcers

**Figure 3:**
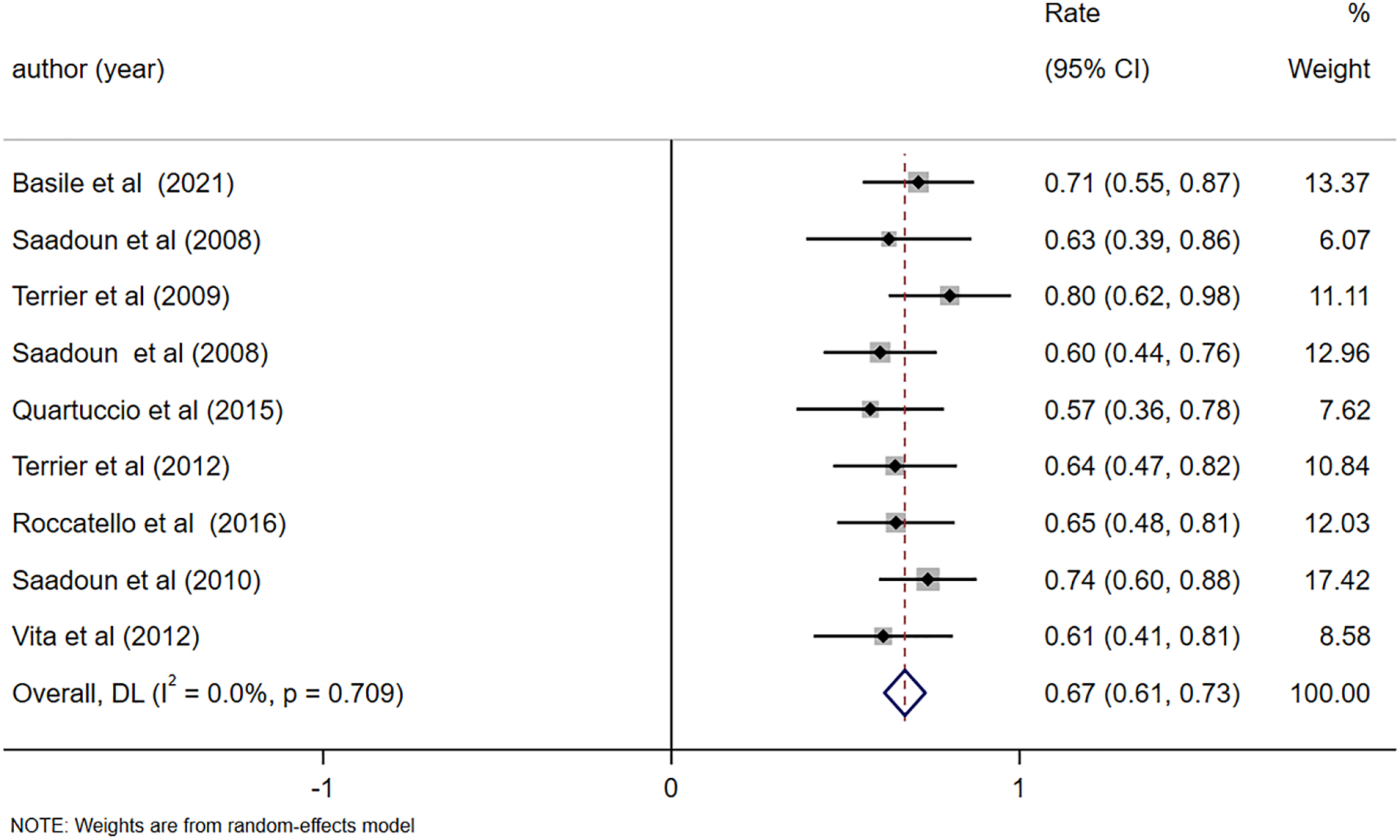
Forest plot of complete clinical response after RTX treatment.

**Figure 4:**
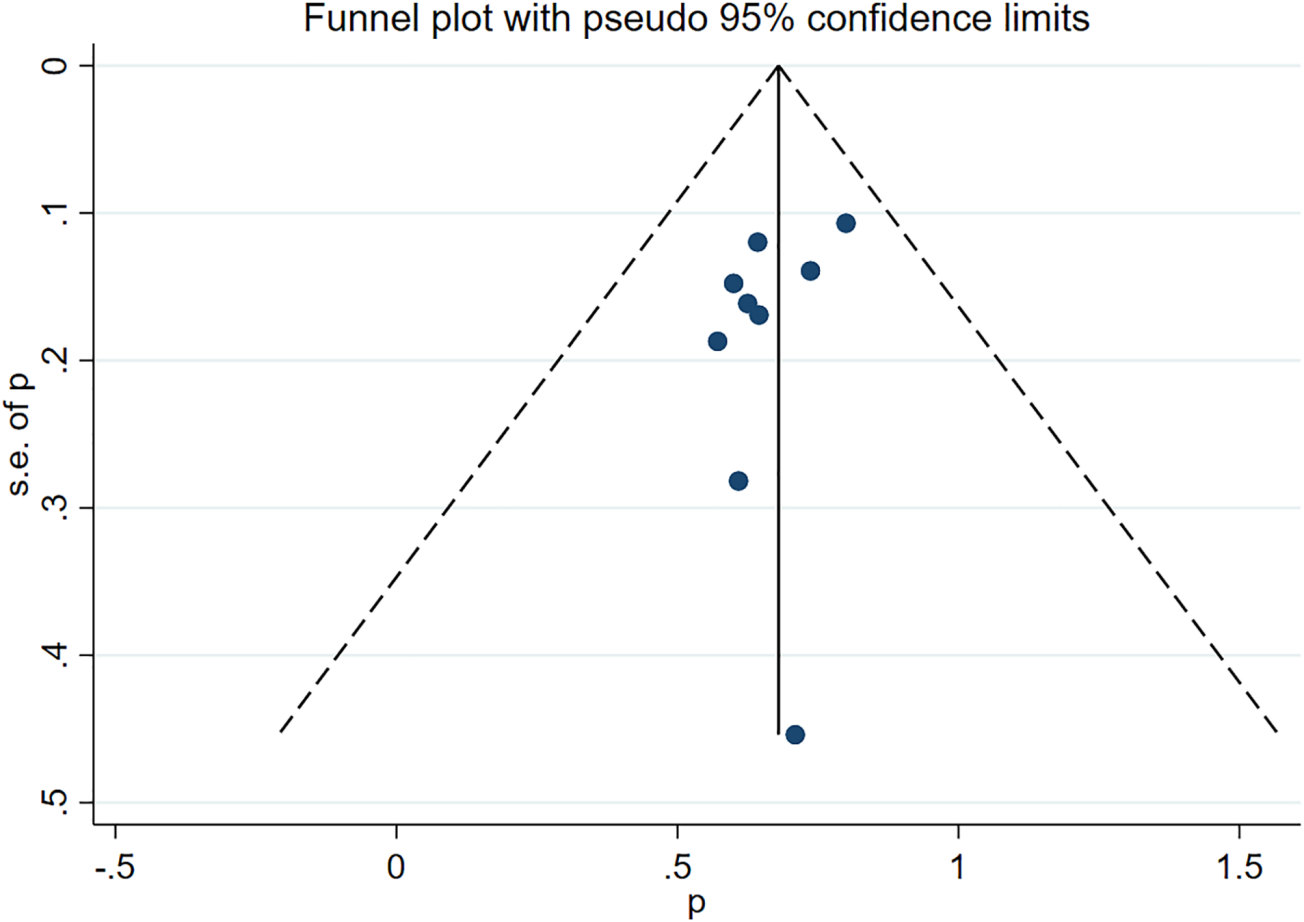
Funnel plot of complete clinical response after RTX treatment.

Nine studies (10, 11, 22–26, 28, 30) reported a complete clinical response, demonstrating the notable efficacy of RTX treatment (Rate = 0.67, 95%CI: 0.61, 0.73, *p* < 0.001). The details are illustrated in Figure 3. Given that the *I*^2^ value was 0.0%, no sensitivity analysis was conducted. The funnel plot showed evidence of asymmetry among the included studies, as depicted in Figure 4. The Egger’s test (*p* = 0.151) indicated the absence of publication bias.

**Figure 5:**
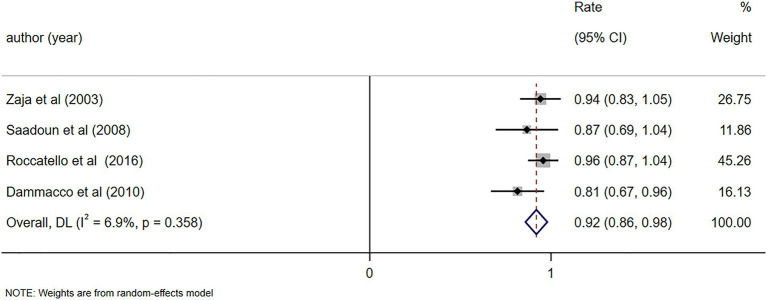
Forest plot of the alleviation rate of symptoms (skin purpura and skin ulcers) after RTX treatment.

The alleviation rate of symptoms such as skin purpura and skin ulcers was reported in four studies (8, 25, 26, 29), demonstrating notable alleviation in symptoms (including skin purpura and skin ulcers) following RTX treatment (Rate = 0.92, 95%CI: 0.86, 0.98, *p* < 0.001). The details are illustrated in Figure 5. Given that the *I*^2^ value was 0.0%, no sensitivity analysis was conducted. The Egger’s test (*p* = 0.140) indicated the absence of publication bias.

### Secondary outcome: laboratory indicator levels

#### C4, IgM, cryoglobulin and RF levels

**Figure 6:**
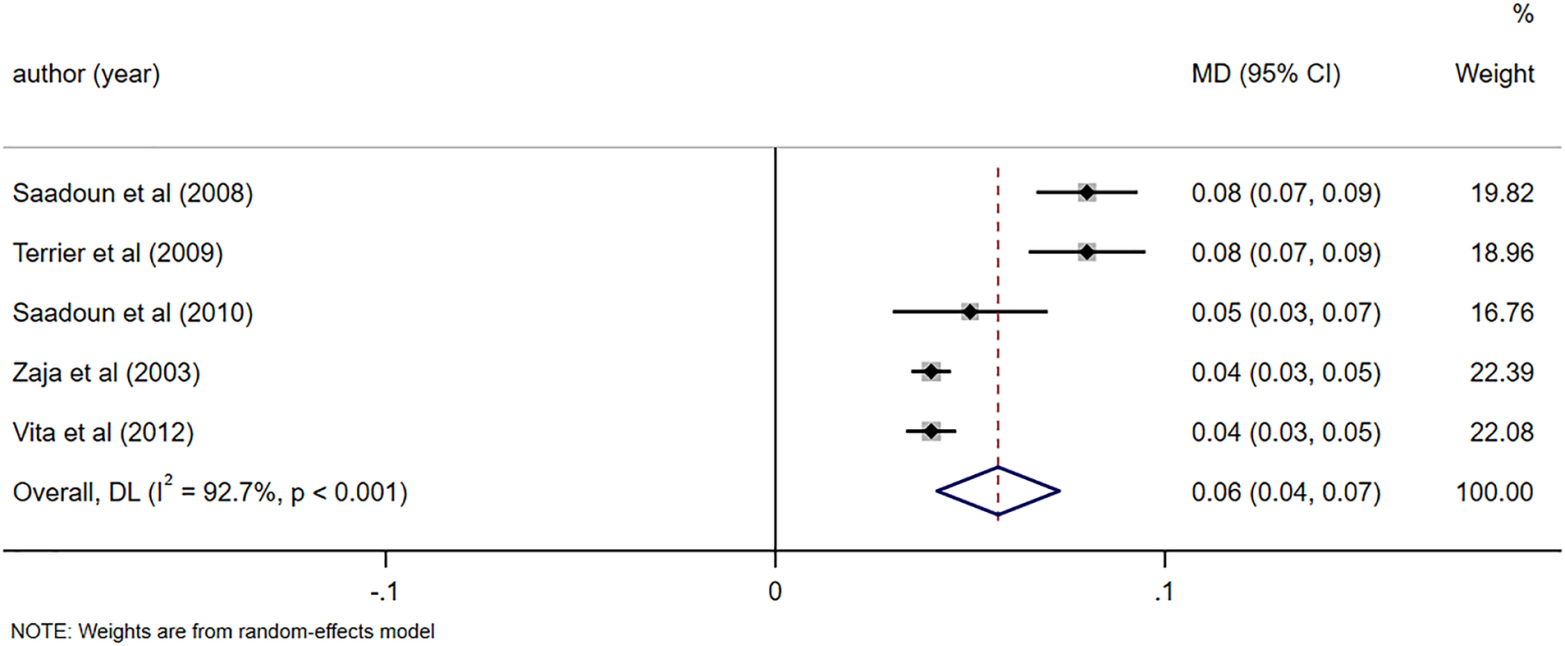
Forest plot of changes in C4 levels (g/L) after RTX treatment.

**Figure 7:**
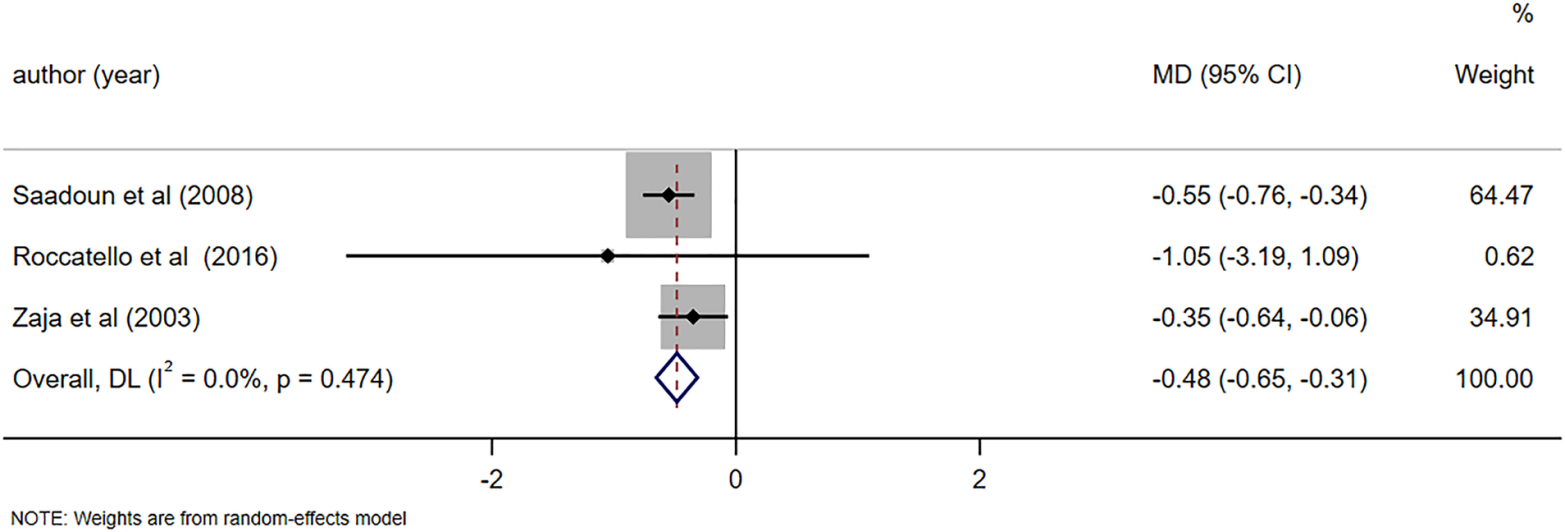
Forest plot of changes in IgM levels (g/L) after RTX treatment.

**Figure 8:**
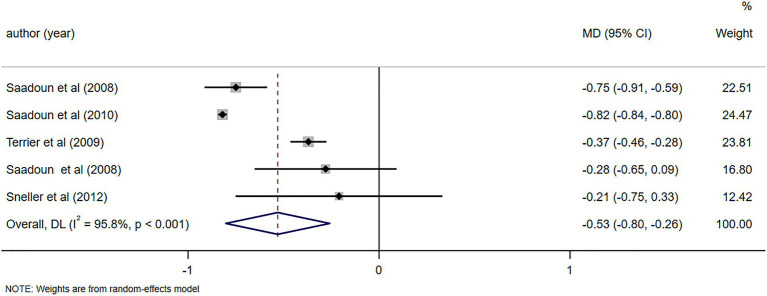
Forest plot of changes in cryoglobulin levels (g/L) after RTX treatment.

**Figure 9:**
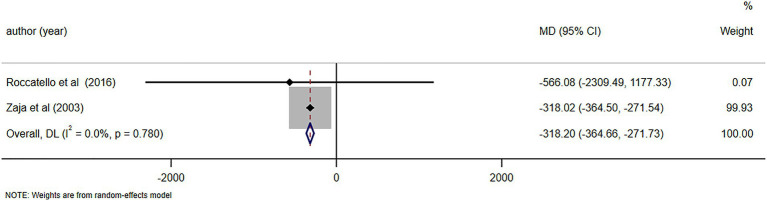
Forest plot of changes in RF levels (IU/ml) after RTX treatment.

C4 levels were reported in five studies (11, 23, 26, 28, 29), demonstrating notable alterations in C4 levels following RTX treatment (MD = 0.06, 95%CI: 0.04, 0.07, *p* < 0.001). The details are illustrated in Figure 6. Due to high heterogeneity (*I*^2^ = 92.7%), a sensitivity analysis was performed to identify the source of heterogeneity, but no clear source was identified (Supplementary Figure S1). The Egger’s test (*p* = 0.112) indicated the absence of publication bias. IgM levels were reported in three studies (10, 25, 29), demonstrating notable alterations in IgM levels following RTX treatment (MD = -0.48, 95%CI: −0.65, −0.31, *p* < 0.001). The details are illustrated in Figure 7. Given that the *I*^2^ value was 0.0%, no sensitivity analysis was conducted. The Egger’s test (*p* = 0.870) indicated the absence of publication bias. Cryoglobulin levels were reported in five studies (10, 11, 26–28), demonstrating notable alterations in cryoglobulin levels following RTX treatment (MD = −0.53, 95%CI: −0.80, −0.26, *p* < 0.001). The details are illustrated in Figure 8. Due to high heterogeneity (*I*^2^ = 95.80%), a sensitivity analysis was performed to identify the source of heterogeneity, but no clear source was identified (Supplementary Figure S2). The Egger’s test (*p* = 0.195) indicated the absence of publication bias. RF levels were reported in three studies (23, 25, 29), demonstrating notable alterations in RF levels following RTX treatment (MD = −318.20, 95%CI: −364.66, −271.73, *p* < 0.001). The details are illustrated in Figure 9. Due to the limited size of literature, no sensitivity and publication bias assessment was performed.

**Figure 10:**
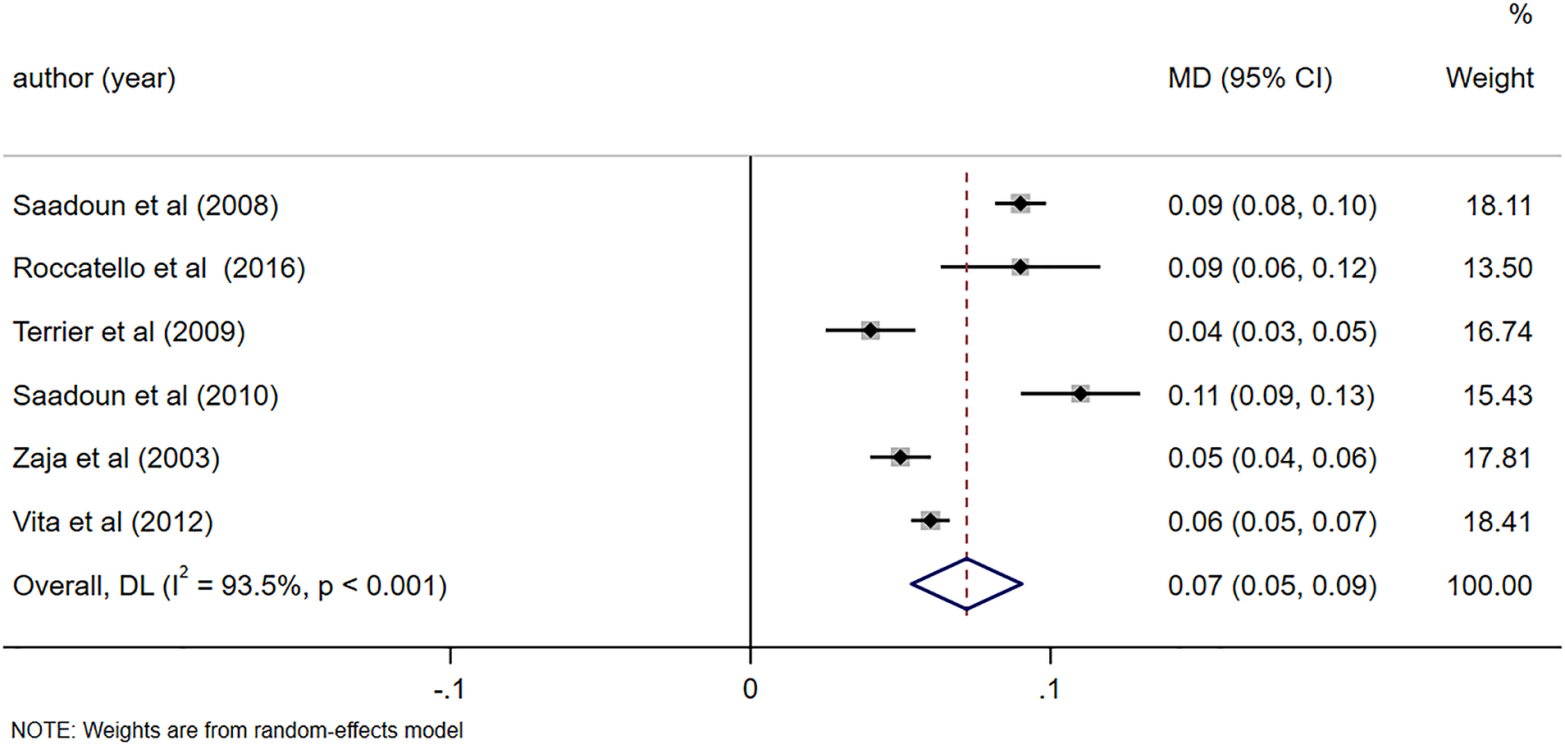
Forest plot of changes in C4 levels (g/L) after a 6-month follow-up.

**Figure 11:**
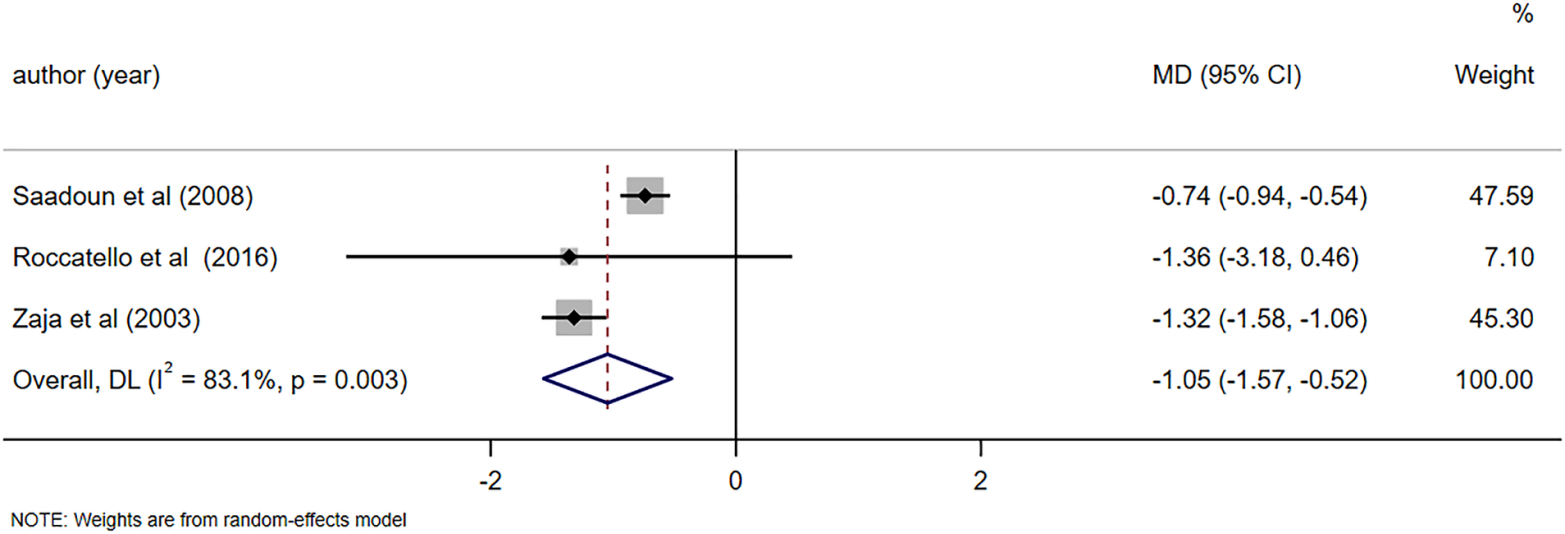
Forest plot of changes in IgM levels (g/L) after a 6-month follow-up.

**Figure 12:**
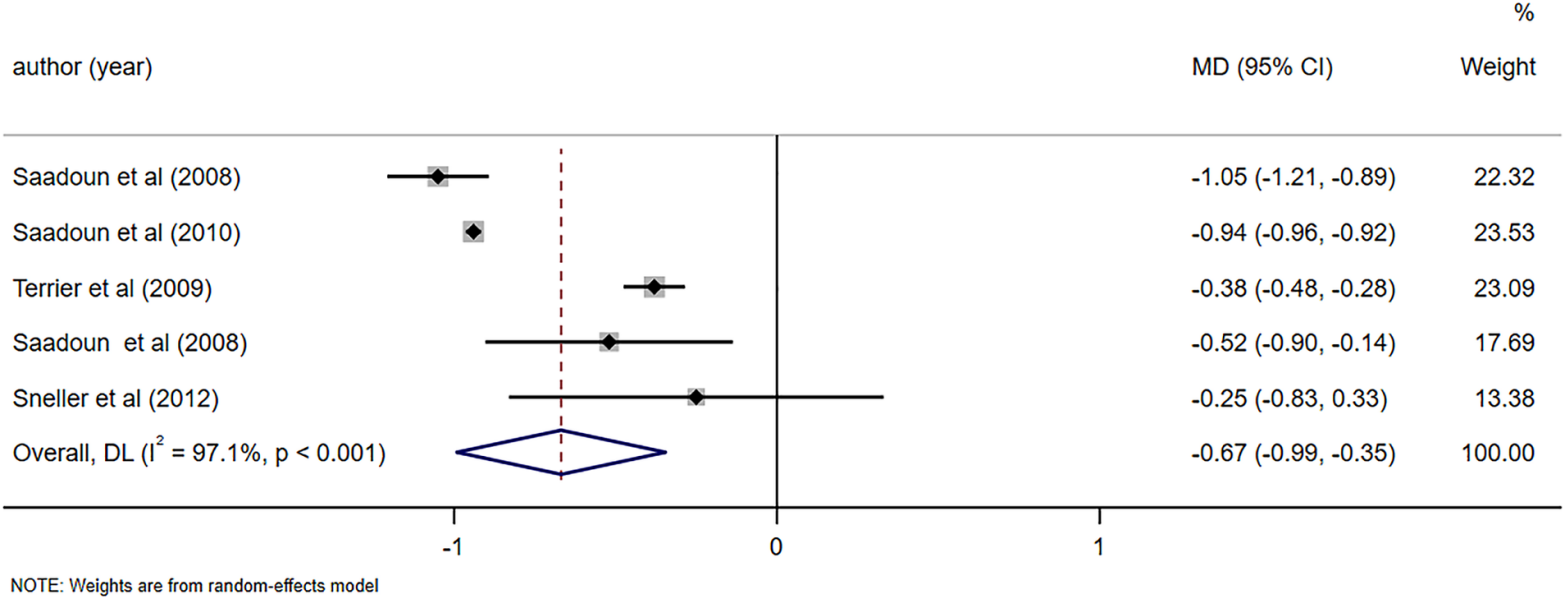
Forest plot of changes in cryoglobulin levels (g/L) after a 6-month follow-up.

**Figure 13:**
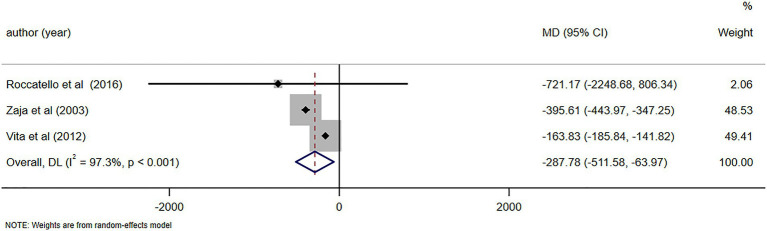
Forest plot of changes in RF levels (IU/ml) after a 6-month follow-up.

Notable alterations in C4 levels were observed following a 6-month follow-up (MD = 0.07, 95%CI: 0.05, 0.09, *p* < 0.001). Further details can be found in Figure 10. In light of the high heterogeneity (*I*^2^ = 93.5%), a sensitivity analysis was performed to identify the source of heterogeneity. However, no clear source was found (Supplementary Figure S3). The Egger’s test (*p* = 0.642) indicated the absence of publication bias. Notable alterations in IgM levels were observed following a 6-month follow-up (MD = −1.05, 95%CI: −1.57, −0.52, *p* < 0.001). Further details can be found in Figure 11. Due to substantial heterogeneity (*I*^2^ = 83.1%), a sensitivity analysis was performed to identify the source of heterogeneity. Despite this, no definitive source of heterogeneity was identified (Supplementary Figure S4). The Egger’s test (*p* = 0.786) indicated the absence of publication bias. Notable alterations in cryoglobulin levels were observed following a 6-month follow-up (MD = −0.67, 95%CI: −0.99, −0.35, *p* < 0.001). Further details can be found in Figure 12. Due to high heterogeneity (*I*^2^ = 97.1%), a sensitivity analysis was performed to identify the source of heterogeneity, but no clear source was identified (Supplementary Figure S5). The Egger’s test (*p* = 0.358) indicated the absence of publication bias. Notable alterations in RF levels were observed following a 6-month follow-up (MD = −287.78, 95%CI: −511.58, −63.97, *p* = 0.012). Further details can be found in Figure 13. Given the high heterogeneity (*I*^2^ = 97.3%), a sensitivity analysis was performed to identify the source of heterogeneity. The analysis failed to identify a clear source of the heterogeneity (Supplementary Figure S6). The Egger’s test (*p* = 0.653) indicated the absence of publication bias.

Notable alterations in C4 levels were observed following a 12-month follow-up (MD = 0.07, 95%CI: 0.03, 0.11, *p* = 0.001). Further details can be found in Supplementary Figure S7. Due to high heterogeneity (*I*^2^ = 93.4%), a sensitivity analysis was performed to identify the source of heterogeneity. However, no specific source could be identified (Supplementary Figure S8). The Egger’s test (*p* = 0.566) indicated the absence of publication bias. Notable alterations in IgM levels were observed following a 12-month follow-up (MD = −0.59, 95%CI: −0.80, −0.38, *p* < 0.001). Further details can be found in Supplementary Figure S9. Due to the limited number of literature, no sensitivity and publication bias assessment was performed. Notable alterations in cryoglobulin levels were observed following a 12-month follow-up (MD = −0.67, 95%CI: −1.15, −0.19, *p* = 0.006). Further details can be found in Figure S10. Due to high heterogeneity (*I*^2^ = 98.3%), a sensitivity analysis was performed to identify the source of heterogeneity. Despite this, no definitive source of heterogeneity was identified (Supplementary Figure S11). The Egger’s test (*p* = 0.534) indicated the absence of publication bias.

## Discussion

To our knowledge, this is the first meta-analysis to evaluate the efficacy of RTX therapy for CV, incorporating data from 5 eligible RCTs and 7 cohort studies. The Meta-analysis results indicated that RTX demonstrated favorable clinical efficacy in treating patients with CV, effectively alleviating clinical symptoms and significantly improving laboratory indicators.

### Therapeutic effect of RTX

The primary measures for assessing clinical remission in patients with CV encompass improvements in clinical manifestations (such as alleviation of skin symptoms, reduced joint pain, and resolution of peripheral neuropathy) as well as normalization of laboratory indices (including decreased serum cryoglobulin levels, lower IgM levels, recovery of serum complement C4, and restoration of hepatic and renal function) (31). Following RTX treatment for CV, fluctuations in IgM levels can be indirectly reflected by dynamic changes in cryoglobulin levels. Numerous clinical studies indicate that cryoglobulin levels generally start to decrease 4–8 weeks post-RTX treatment, reaching their lowest point between 12 and 24 weeks. In patients with HCV-related mixed cryoglobulinemia, the viral infection may initially obscure a significant decrease in IgM levels (1). In addition, a randomized controlled retrospective study showed that (32) clinical symptoms were significantly relieved in 65–80% of CV patients after the administration of RTX. Laboratory indicators such as cryoglobulin and RF also significantly decreased. Moreover, serum C4 levels returned to normal. In a 2023 systematic review by Covic et al. (33), it was concluded that RTX-based therapy, either as monotherapy or in combination, demonstrated therapeutic benefits for patients with hepatitis C-associated CV, resulting in high rates of complete clinical response.

### Heterogeneity in RTX efficacy for patients with or without HCV infection

In CV patients with without concomitant HCV infection, RTX in combination with glucocorticoids is often employed. This regimen demonstrates the most pronounced clinical, renal, and immunological benefits, as well as therapeutic efficacy (30, 34). For CV patients with concomitant HCV infection, antiviral drugs are usually incorporated into the treatment protocol for combined therapy (8, 35, 36). RTX demonstrates favorable therapeutic effects in both patient groups. However, relapse is more frequently observed in HCV-positive patients, typically due to suboptimal virological control (28). Conversely, in CV patients without HCV infection, there is an increased risk of serious infections, often associated with high-dose corticosteroid use (30).

### Mechanism of RTX therapy for CV

These findings align with our meta-analysis, which demonstrates the beneficial effects of RTX in treating CV patients, specifically in terms of clinical response rates, symptom alleviation, and improved laboratory parameters. This may be due to the targeted induction of the apoptosis of B cells by RTX, which may reduce the production of autoantibodies. In addition, B cells can produce cytokines, participate in inflammatory responses, and enhance the response of T cells (37). RTX can consume amplified and activated CD20-positive B cells in CV patients. Some studies have suggested that the depletion of B cells may result in the activation of T cells (12). Mathur et al. (38) observed a correlation of RTX therapy with the improvement in HCV-specific T cell function across individuals with HCV-MC vasculitis. Research findings suggest that cell depletion therapy restores T cell function and reverses exhaustion in individuals with HCV-associated CV. This provides new perspectives on the interplay between B cells and T cells in the development of HCV-MC vasculitis (38).

### Safety and tolerance of RTX and re-treatment strategies

The RTX has been proven to be effective and safe in the treatment of CV. However, it should be noted that its safety warrants further investigation. Adverse reactions such as chills, arrhythmia, and hypersensitivity reactions may occur during RTX administration, typically during the initial infusion. These symptoms generally resolve with symptomatic treatment (39). Some studies have reported hypotension during RTX infusion. It is suggested to have slow infusion rates and temporary cessation of antihypertensive agents during RTX administration (40). Before the introduction of RTX, liver failure and infection were the leading causes of mortality in CV patients (41). In cases where the severity of the disease is comparable, the 5-year survival for patients receiving RTX treatment is 75%. About 60% of patients remained relapse-free without requiring any treatment within 10 years. Among patients who experience a relapse, the probability of remaining asymptomatic for 5 years following the initiation of similar treatment is 80%, with no observed elevation in the incidence of severe infections or hepatic failure (25).

Although RTX demonstrates favorable clinical efficacy, its therapeutic effects are temporary. Retrospective studies have reported the exacerbation of vasculitis and the occurrence of severe infections in CV patients following RTX treatment. Desbois et al. (42) that among 185 CV patients, 7 patients (3.4%) experienced recurrence, all of which were caused by type II mixed cryoglobulinemia vasculitis. The recurrence occurred at a median time (2–16 days) after the infusion of RTX (43). Among the 64 reported CV patients, 14 (22%) receiving RTX treatment experienced worsening of their condition, with a median time to worsening of approximately 5.5 days following RTX administration. The deterioration often occurred between 2 days and 1 week following treatment. The affected organ systems mainly included the skin, kidneys, and peripheral nerves, and the condition was more likely to occur in patients with underlying B-cell lymphoproliferative disorders.

Following RTX treatment failure, diverse therapeutic approaches were utilized, such as glucocorticoids (GC), alkylating agents, RTX in combination with other therapies, and belimumab. Specifically, the highest clinical response rates were observed with the combinations of anti-CD20 plus belimumab (100%), alkylating agents alone (82%), and anti-CD20 plus alkylating agents (73%) (44). Some studies have also indicated that in essential (EM) and connective tissue disease (CTD)-related mixed CV patients who relapsed after initial RTX treatment, subsequent RTX maintenance therapy reduced the recurrence rate (45). For patients with primary cryoglobulinemia, treatment should include GCs in combination with RTX. A rapid and progressive reduction of GC dosage is crucial to minimize the likelihood of infectious complications (46). For patients experiencing relapse of HCV-related CV, antiviral therapy should be continued or restarted. This should be combined with immunosuppressive agents, such as RTX and cyclophosphamide, to effectively control the patient’s symptoms.

### Different doses of RTX in the treatment of CV

Most studies have adopted the standard RTX dosage of 375 mg/m^2^ administered weekly for four doses, a regimen derived from lymphoma treatment. However, some research has explored alternative dosages, including both higher and lower regimens. Colantuono et al. (39) treated 37 patients with refractory mixed CV using a low-dose RTX regimen (250 mg/m^2^ weekly for 2 weeks), achieving an 80% response rate and a 68% complete remission rate. The findings demonstrated that repeated administration of a low-dose RTX regimen for relapsed mixed cryoglobulinemia is an effective, safe, and cost-effective strategy for its long-term disease management (39). A separate observational study utilized the same low-dose regimen in 31 patients with refractory mixed CV, resulting in clinical responses in 22 patients, achieving a response rate of 70.96% (22). For the induction of remission, high-dose RTX protocols predominantly utilize a dosage of 375 mg/m^2^ administered once weekly for four consecutive weeks. This is followed by a maintenance dose of 500 mg every 6–9 months (47). This protocol is primarily indicated for patients presenting with severe clinical manifestations. Future efforts may focus on optimizing RTX dosing regimens, for instance, by comparing high-dose and low-dose strategies in CV patients.

The included studies were of moderate to high quality, indicating the robustness of our analysis findings. Data were pooled from multiple studies to enhance the power to detect the efficacy of RTX. This study aimed to contribute to the body of evidence-based medicine, particularly in situations where individual studies are limited by small sample sizes or fail to demonstrate statistically significant effects. This study also has several limitations. Firstly, the included studies were predominantly from Italy (90%), with only one study from elsewhere. This is likely due to the fact that Italy has several world-renowned hematology research and clinical centers. Italy was an important clinical trial center for early RTX clinical trials involving multi-national European recruitment, and this has provided Italian researchers with a wealth of clinical data and opportunities for publication. Secondly, the limited number (*n* = 12) and the quality heterogeneity of the included studies may introduce bias into the meta-analysis results. Thirdly, there is a lack of blank controls for RTX in the included studies. Due to the fact that some studies compared the clinical efficacy of RTX monotherapy versus RTX combined with other antiviral agents for CV treatment, while others solely investigated the clinical efficacy of RTX monotherapy. In addition, several studies had incomplete control groups with only baseline data and no follow-up. This lack of data made it difficult to extract endpoint data for blank controls. Therefore, a direct comparison of the effectiveness of RTX in treating CV patients is not feasible without a control group. Fourthly, due to the limitations of the included data, the follow-up durations for the same or different outcome measures varied across studies. Some studies did not provide initial treatment data, while others lacked subsequent follow-up data, such as 6-month or 12-month follow-up data. Consequently, the follow-up durations vary among studies, complicating the extraction of outcome metrics and the performance of comprehensive subgroup analyses. This variability could potentially impact the final outcomes. Lastly, MC involves not only skin-related symptoms but also more severe manifestations affecting the kidneys and peripheral nervous system. However, a paucity of extractable outcome data pertaining to improvements in renal and peripheral neuropathy symptoms led us to analyze only the remission of skin-related symptoms as an outcome measure. This limitation might lead to a lack of comprehensive analysis in our results.

## Conclusion

To sum up, RTX has shown promising application prospects in the management of CV. However, more extensive research is essential to explore the long-term efficacy, optimal dosage, and combination strategies with other drugs. Owing to the limited number and quality of existing studies, future research necessitates high-quality, large-scale, double-blind RCTs to validate these findings.

## Data Availability

The original contributions presented in the study are included in the article/Supplementary material, further inquiries can be directed to the corresponding author.
