# Supplementary material for: Meta-analysis of the efficacy of rituximab in the management of cryoglobulinemic vasculitis
Source: Front Med (Lausanne). 2025 Aug 29;12:1591366. doi: 10.3389/fmed.2025.1591366 (PMC12426258; doi:10.3389/fmed.2025.1591366)
Supplement: Supplementary file 1 [file Table_1.docx]

Table S1 Literature search strategy of PubMed

| Search number | Query | Records |
| --- | --- | --- |
| #1 | "Vasculitis"[Mesh] | 107292 |
| #2 | (((((((((((((((((Vasculitis[Title/Abstract]) OR (Vasculitides[Title/Abstract])) OR (Angiitis[Title/Abstract])) OR (Angiitides[Title/Abstract])) OR (acute vasculitis[Title/Abstract])) OR (angiitic lesions[Title/Abstract])) OR (angitis[Title/Abstract])) OR (blood vessel inflammation[Title/Abstract])) OR (obliterating vasculitis[Title/Abstract])) OR (vascular inflammation[Title/Abstract])) OR (vasculitic inflammation[Title/Abstract])) OR (vasculitic inflammatory disease[Title/Abstract])) OR (vasculitic inflammatory disorder[Title/Abstract])) OR (vasculitic inflammatory lesion[Title/Abstract])) OR (vasculitic lesion[Title/Abstract])) OR (vasculitic lesions[Title/Abstract])) OR (vasculitic syndrome[Title/Abstract])) OR (vessel inflammation[Title/Abstract]) | 52697 |
| #3 | ("Vasculitis"[Mesh]) OR ((((((((((((((((((Vasculitis[Title/Abstract]) OR (Vasculitides[Title/Abstract])) OR (Angiitis[Title/Abstract])) OR (Angiitides[Title/Abstract])) OR (acute vasculitis[Title/Abstract])) OR (angiitic lesions[Title/Abstract])) OR (angitis[Title/Abstract])) OR (blood vessel inflammation[Title/Abstract])) OR (obliterating vasculitis[Title/Abstract])) OR (vascular inflammation[Title/Abstract])) OR (vasculitic inflammation[Title/Abstract])) OR (vasculitic inflammatory disease[Title/Abstract])) OR (vasculitic inflammatory disorder[Title/Abstract])) OR (vasculitic inflammatory lesion[Title/Abstract])) OR (vasculitic lesion[Title/Abstract])) OR (vasculitic lesions[Title/Abstract])) OR (vasculitic syndrome[Title/Abstract])) OR (vessel inflammation[Title/Abstract])) | 132202 |
| #4 | "Rituximab"[Mesh] | 20157 |
| #5 | ((((((((((((((((((((((((((((((((((((Rituximab[Title/Abstract]) OR (Mabthera[Title/Abstract])) OR (IDEC-C2B8 Antibody[Title/Abstract])) OR (IDEC C2B8 Antibody[Title/Abstract])) OR (IDEC-C2B8[Title/Abstract])) OR (IDEC C2B8[Title/Abstract])) OR (GP2013[Title/Abstract])) OR (Rituxan[Title/Abstract])) OR (Acellbia[Title/Abstract])) OR (Blitzima[Title/Abstract])) OR (Cimabior[Title/Abstract])) OR (Halpryza[Title/Abstract])) OR (Kikuzubam[Title/Abstract])) OR (Mabthera[Title/Abstract])) OR (Redditux[Title/Abstract])) OR (Reditux[Title/Abstract])) OR (Retuxira[Title/Abstract])) OR (Riabni[Title/Abstract])) OR (Ristova[Title/Abstract])) OR (Ritemvia[Title/Abstract])) OR (Ritucad[Title/Abstract])) OR (Ritumax[Title/Abstract])) OR (rituximab abbs[Title/Abstract])) OR (rituximab arrx[Title/Abstract])) OR (rituximab pvvr[Title/Abstract])) OR (rituximab-abbs[Title/Abstract])) OR (rituximab-arrx[Title/Abstract])) OR (rituximab-pvvr[Title/Abstract])) OR (rituxin[Title/Abstract])) OR (rituzena[Title/Abstract])) OR (rixathon[Title/Abstract])) OR (riximyo[Title/Abstract])) OR (ruxience[Title/Abstract])) OR (tidecron[Title/Abstract])) OR (truxima[Title/Abstract])) OR (tuxella[Title/Abstract])) OR (zytux[Title/Abstract]) | 30406 |
| #6 | ("Rituximab"[Mesh]) OR (((((((((((((((((((((((((((((((((((((Rituximab[Title/Abstract]) OR (Mabthera[Title/Abstract])) OR (IDEC-C2B8 Antibody[Title/Abstract])) OR (IDEC C2B8 Antibody[Title/Abstract])) OR (IDEC-C2B8[Title/Abstract])) OR (IDEC C2B8[Title/Abstract])) OR (GP2013[Title/Abstract])) OR (Rituxan[Title/Abstract])) OR (Acellbia[Title/Abstract])) OR (Blitzima[Title/Abstract])) OR (Cimabior[Title/Abstract])) OR (Halpryza[Title/Abstract])) OR (Kikuzubam[Title/Abstract])) OR (Mabthera[Title/Abstract])) OR (Redditux[Title/Abstract])) OR (Reditux[Title/Abstract])) OR (Retuxira[Title/Abstract])) OR (Riabni[Title/Abstract])) OR (Ristova[Title/Abstract])) OR (Ritemvia[Title/Abstract])) OR (Ritucad[Title/Abstract])) OR (Ritumax[Title/Abstract])) OR (rituximab abbs[Title/Abstract])) OR (rituximab arrx[Title/Abstract])) OR (rituximab pvvr[Title/Abstract])) OR (rituximab-abbs[Title/Abstract])) OR (rituximab-arrx[Title/Abstract])) OR (rituximab-pvvr[Title/Abstract])) OR (rituxin[Title/Abstract])) OR (rituzena[Title/Abstract])) OR (rixathon[Title/Abstract])) OR (riximyo[Title/Abstract])) OR (ruxience[Title/Abstract])) OR (tidecron[Title/Abstract])) OR (truxima[Title/Abstract])) OR (tuxella[Title/Abstract])) OR (zytux[Title/Abstract])) | 34083 |
| #7 | (("Vasculitis"[Mesh]) OR ((((((((((((((((((Vasculitis[Title/Abstract]) OR (Vasculitides[Title/Abstract])) OR (Angiitis[Title/Abstract])) OR (Angiitides[Title/Abstract])) OR (acute vasculitis[Title/Abstract])) OR (angiitic lesions[Title/Abstract])) OR (angitis[Title/Abstract])) OR (blood vessel inflammation[Title/Abstract])) OR (obliterating vasculitis[Title/Abstract])) OR (vascular inflammation[Title/Abstract])) OR (vasculitic inflammation[Title/Abstract])) OR (vasculitic inflammatory disease[Title/Abstract])) OR (vasculitic inflammatory disorder[Title/Abstract])) OR (vasculitic inflammatory lesion[Title/Abstract])) OR (vasculitic lesion[Title/Abstract])) OR (vasculitic lesions[Title/Abstract])) OR (vasculitic syndrome[Title/Abstract])) OR (vessel inflammation[Title/Abstract]))) AND (("Rituximab"[Mesh]) OR (((((((((((((((((((((((((((((((((((((Rituximab[Title/Abstract]) OR (Mabthera[Title/Abstract])) OR (IDEC-C2B8 Antibody[Title/Abstract])) OR (IDEC C2B8 Antibody[Title/Abstract])) OR (IDEC-C2B8[Title/Abstract])) OR (IDEC C2B8[Title/Abstract])) OR (GP2013[Title/Abstract])) OR (Rituxan[Title/Abstract])) OR (Acellbia[Title/Abstract])) OR (Blitzima[Title/Abstract])) OR (Cimabior[Title/Abstract])) OR (Halpryza[Title/Abstract])) OR (Kikuzubam[Title/Abstract])) OR (Mabthera[Title/Abstract])) OR (Redditux[Title/Abstract])) OR (Reditux[Title/Abstract])) OR (Retuxira[Title/Abstract])) OR (Riabni[Title/Abstract])) OR (Ristova[Title/Abstract])) OR (Ritemvia[Title/Abstract])) OR (Ritucad[Title/Abstract])) OR (Ritumax[Title/Abstract])) OR (rituximab abbs[Title/Abstract])) OR (rituximab arrx[Title/Abstract])) OR (rituximab pvvr[Title/Abstract])) OR (rituximab-abbs[Title/Abstract])) OR (rituximab-arrx[Title/Abstract])) OR (rituximab-pvvr[Title/Abstract])) OR (rituxin[Title/Abstract])) OR (rituzena[Title/Abstract])) OR (rixathon[Title/Abstract])) OR (riximyo[Title/Abstract])) OR (ruxience[Title/Abstract])) OR (tidecron[Title/Abstract])) OR (truxima[Title/Abstract])) OR (tuxella[Title/Abstract])) OR (zytux[Title/Abstract]))) | 2387 |
| #8 | (Cryoglobulinemic[Title/Abstract]) OR (Cryoglobulinemia[Title/Abstract]) | 3957 |
| #9 | ((("Vasculitis"[Mesh]) OR ((((((((((((((((((Vasculitis[Title/Abstract]) OR (Vasculitides[Title/Abstract])) OR (Angiitis[Title/Abstract])) OR (Angiitides[Title/Abstract])) OR (acute vasculitis[Title/Abstract])) OR (angiitic lesions[Title/Abstract])) OR (angitis[Title/Abstract])) OR (blood vessel inflammation[Title/Abstract])) OR (obliterating vasculitis[Title/Abstract])) OR (vascular inflammation[Title/Abstract])) OR (vasculitic inflammation[Title/Abstract])) OR (vasculitic inflammatory disease[Title/Abstract])) OR (vasculitic inflammatory disorder[Title/Abstract])) OR (vasculitic inflammatory lesion[Title/Abstract])) OR (vasculitic lesion[Title/Abstract])) OR (vasculitic lesions[Title/Abstract])) OR (vasculitic syndrome[Title/Abstract])) OR (vessel inflammation[Title/Abstract]))) AND (("Rituximab"[Mesh]) OR (((((((((((((((((((((((((((((((((((((Rituximab[Title/Abstract]) OR (Mabthera[Title/Abstract])) OR (IDEC-C2B8 Antibody[Title/Abstract])) OR (IDEC C2B8 Antibody[Title/Abstract])) OR (IDEC-C2B8[Title/Abstract])) OR (IDEC C2B8[Title/Abstract])) OR (GP2013[Title/Abstract])) OR (Rituxan[Title/Abstract])) OR (Acellbia[Title/Abstract])) OR (Blitzima[Title/Abstract])) OR (Cimabior[Title/Abstract])) OR (Halpryza[Title/Abstract])) OR (Kikuzubam[Title/Abstract])) OR (Mabthera[Title/Abstract])) OR (Redditux[Title/Abstract])) OR (Reditux[Title/Abstract])) OR (Retuxira[Title/Abstract])) OR (Riabni[Title/Abstract])) OR (Ristova[Title/Abstract])) OR (Ritemvia[Title/Abstract])) OR (Ritucad[Title/Abstract])) OR (Ritumax[Title/Abstract])) OR (rituximab abbs[Title/Abstract])) OR (rituximab arrx[Title/Abstract])) OR (rituximab pvvr[Title/Abstract])) OR (rituximab-abbs[Title/Abstract])) OR (rituximab-arrx[Title/Abstract])) OR (rituximab-pvvr[Title/Abstract])) OR (rituxin[Title/Abstract])) OR (rituzena[Title/Abstract])) OR (rixathon[Title/Abstract])) OR (riximyo[Title/Abstract])) OR (ruxience[Title/Abstract])) OR (tidecron[Title/Abstract])) OR (truxima[Title/Abstract])) OR (tuxella[Title/Abstract])) OR (zytux[Title/Abstract])))) AND ((Cryoglobulinemic[Title/Abstract]) OR (Cryoglobulinemia[Title/Abstract])) | 223 |
