# Supplementary material for: Meta-analysis of the efficacy of rituximab in the management of cryoglobulinemic vasculitis
Source: Front Med (Lausanne). 2025 Aug 29;12:1591366. doi: 10.3389/fmed.2025.1591366 (PMC12426258; doi:10.3389/fmed.2025.1591366)
Supplement: Supplementary file 3 [file Table_3.docx]

Table S3 Literature search strategy of Cochrane Library

| Search number | Query | Records |
| --- | --- | --- |
| #1 | MeSH descriptor: [Vasculitis] explode all trees | 2581 |
| #2 | vasculitis:ab,ti OR vasculitides:ab,ti OR angiitis:ab,ti OR angiitides:ab,ti OR 'acute vasculitis':ab,ti OR 'angiitic lesions':ab,ti OR angitis:ab,ti OR 'blood vessel inflammation':ab,ti OR 'obliterating vasculitis':ab,ti OR 'vascular inflammation':ab,ti OR 'vasculitic inflammation':ab,ti OR 'vasculitic inflammatory disease':ab,ti OR 'vasculitic inflammatory disorder':ab,ti OR 'vasculitic inflammatory lesion':ab,ti OR 'vasculitic lesion':ab,ti OR 'vasculitic lesions':ab,ti OR 'vasculitic syndrome':ab,ti OR 'vessel inflammation':ab,ti | 5870 |
| #3 | MeSH descriptor: [Rituximab] explode all trees | 2057 |
| #4 | rituximab:ab,ti OR 'idec-c2b8 antibody':ab,ti OR 'idec c2b8 antibody':ab,ti OR 'idec c2b8':ab,ti OR gp2013:ab,ti OR rituxan:ab,ti OR acellbia:ab,ti OR blitzima:ab,ti OR cimabior:ab,ti OR halpryza:ab,ti OR kikuzubam:ab,ti OR mabthera:ab,ti OR redditux:ab,ti OR reditux:ab,ti OR retuxira:ab,ti OR riabni:ab,ti OR ristova:ab,ti OR ritemvia:ab,ti OR ritucad:ab,ti OR ritumax:ab,ti OR 'rituximab abbs':ab,ti OR 'rituximab arrx':ab,ti OR 'rituximab pvvr':ab,ti OR rituxin:ab,ti OR rituzena:ab,ti OR rixathon:ab,ti OR riximyo:ab,ti OR ruxience:ab,ti OR tidecron:ab,ti OR truxima:ab,ti OR tuxella:ab,ti OR zytux:ab,ti | 6218 |
| #5 | #1 or #2 | 7942 |
| #6 | #3 or #4 | 6403 |
| #7 | (Cryoglobulinemic):ti,ab,kw OR (Cryoglobulinemia):ti,ab,kw | 87 |
| #8 | #5 and #7 | 36 |
