# Supplementary material for: Meta-analysis of the efficacy of rituximab in the management of cryoglobulinemic vasculitis
Source: Front Med (Lausanne). 2025 Aug 29;12:1591366. doi: 10.3389/fmed.2025.1591366 (PMC12426258; doi:10.3389/fmed.2025.1591366)
Supplement: Supplementary file 4 [file Table_4.docx]

Table S4 Literature search strategy of Web of Science

| Search number | Query | Records |
| --- | --- | --- |
| #1 | Vasculitis (Topic) OR Vasculitides (Topic) OR Angiitis (Topic) OR Angiitides (Topic) OR acute vasculitis (Topic) OR angiitic lesions (Topic) OR angitis (Topic) OR blood vessel inflammation (Topic) OR obliterating vasculitis (Topic) OR vascular inflammation (Topic) OR vasculitic inflammation (Topic) OR vasculitic inflammatory disease (Topic) OR vasculitic inflammatory disorder (Topic) OR vasculitic inflammatory lesion (Topic) OR vasculitic lesion (Topic) OR vasculitic lesions (Topic) OR vasculitic syndrome (Topic) OR vessel inflammation (Topic) | 417215 |
| #2 | Rituximab (Topic) OR Mabthera (Topic) OR IDEC-C2B8 Antibody (Topic) OR IDEC C2B8 Antibody (Topic) OR IDEC-C2B8 (Topic) OR IDEC C2B8 (Topic) OR GP2013 (Topic) OR Rituxan (Topic) OR Acellbia (Topic) OR Blitzima (Topic) OR Cimabior (Topic) OR Halpryza (Topic) OR Kikuzubam (Topic) OR Mabthera (Topic) OR Redditux (Topic) OR Reditux (Topic) OR Retuxira (Topic) OR Riabni (Topic) OR Ristova (Topic) OR Ritemvia (Topic) OR Ritucad (Topic) OR Ritumax (Topic) OR rituximab abbs (Topic) OR rituximab arrx (Topic) OR rituximab pvvr (Topic) OR rituximab-abbs (Topic) OR rituximab-arrx (Topic) OR rituximab-pvvr (Topic) OR rituxin (Topic) OR rituzena (Topic) OR rixathon (Topic) OR riximyo (Topic) OR ruxience (Topic) OR tidecron (Topic) OR truxima (Topic) OR tuxella (Topic) OR zytux (Topic) | 75994 |
| #3 | Cryoglobulinemic (Topic) OR Cryoglobulinemia (Topic) | 8538 |
| #4 | #3 AND #2 AND #1 | 525 |
