# Supplementary material for: Meta-analysis of the efficacy of rituximab in the management of cryoglobulinemic vasculitis
Source: Front Med (Lausanne). 2025 Aug 29;12:1591366. doi: 10.3389/fmed.2025.1591366 (PMC12426258; doi:10.3389/fmed.2025.1591366)
Supplement: Supplementary file 5 [file Supplementary_file_1.docx]

Figure S1. Sensitivity analysis of changes in C4 level after RTX treatment

Figure S2. Sensitivity analysis of changes in cryoglobulin level after RTX treatment

Figure S3. Sensitivity analysis of changes in C4 level after 6 months of follow-up

Figure S4. Sensitivity analysis of changes in IgM level after 6 months of follow-up

Figure S5. Sensitivity analysis of changes in cryoglobulin level after 6 months of follow-up

Figure S6. Sensitivity analysis of changes in RF level after 6 months of follow-up
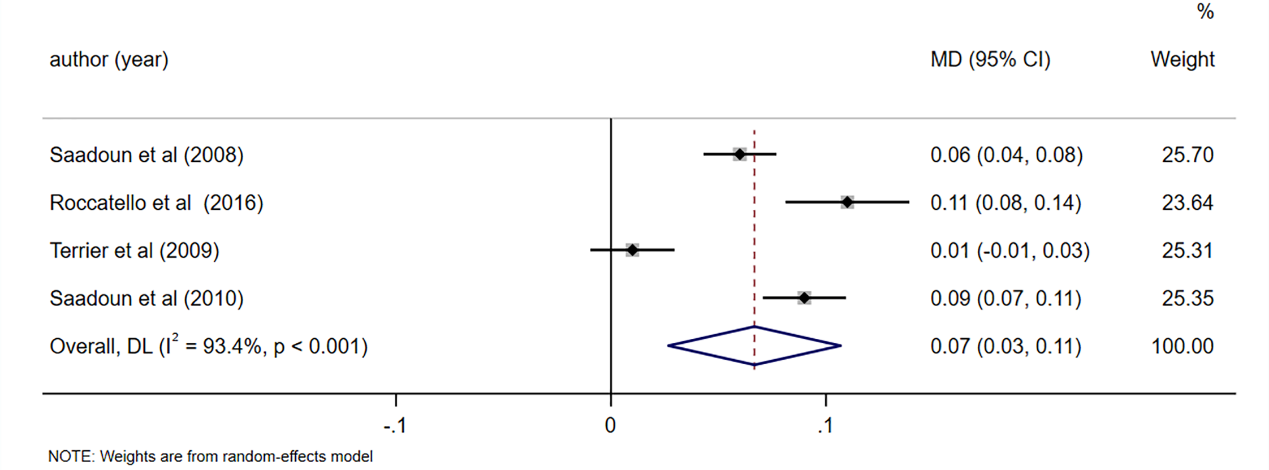


Figure S7 Forest plot of changes in C4 levels (g/L) after a 12-month follow-up

Figure S8. Sensitivity analysis of changes in C4 level after 12 months of follow-up
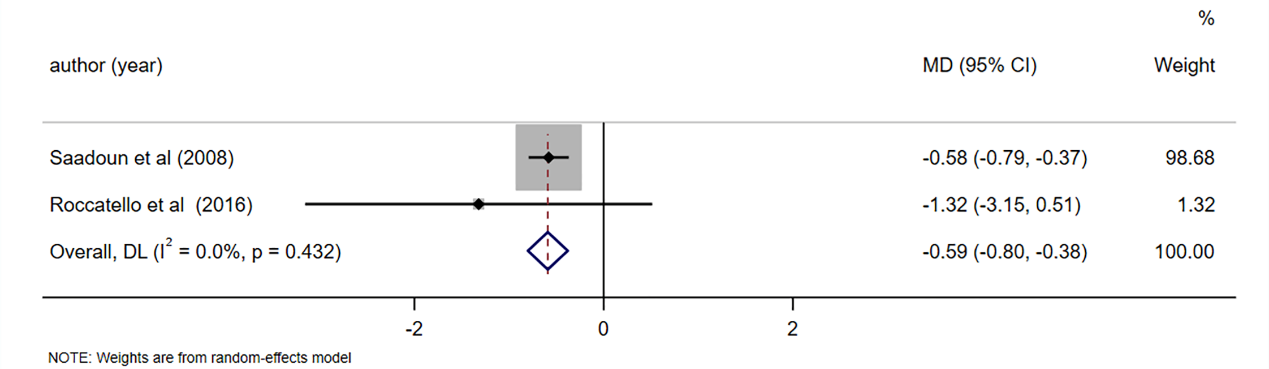


Figure S9 Forest plot of changes in IgM levels (g/L) after a 12-month follow-up


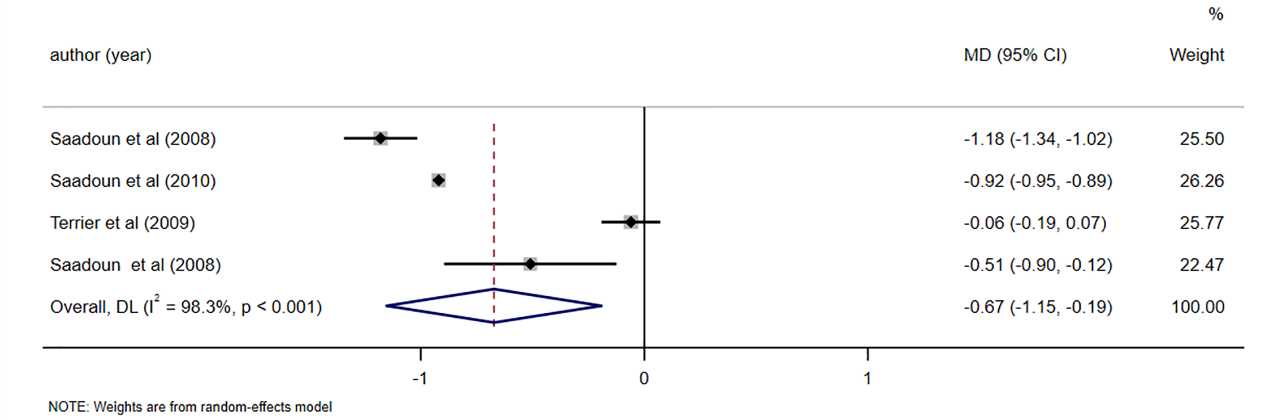


Figure S10 Forest plot of changes in cryoglobulin levels (g/L) after a 12-month

Figure S11. Sensitivity analysis of changes in cryoglobulin level after 12 months of follow-up
